# Bioactive Dental Materials: The Current Status

**DOI:** 10.3390/ma15062016

**Published:** 2022-03-09

**Authors:** Gianrico Spagnuolo

**Affiliations:** Department of Neurosciences, Reproductive and Odontostomatological Sciences, University of Naples “Federico II”, 80138 Napoli, Italy; gspagnuo@unina.it

The field of dental materials has undergone a significant evolution in recent years. From restorative and endodontic materials to bioactive agents used for bone reconstruction, the introduction of newer techniques and materials has changed dental practice and treatment planning [1,2,3]. In modern dentistry, there is a great interest in the application of “bioactive” materials for restorative and reconstructive purposes. It must be noted that depending on the application, the perception of what is actually considered “bioactive” differs. In restorative dentistry, the term bioactive usually refers to the ability of a material to form hydroxyapatite crystals on its surface. In implantology, bioactivity concerns the potential of some materials, such as calcium phosphate ceramics and glasses, to provide a direct chemical bond between the implant and the recipient bone. In preventive dentistry, bioactive toothpastes have been employed with the aim to remineralize the outer enamel surface [4]. However, from a biological perspective, bioactive compounds are considered as agents that potentially interact—in a positive way—with living cells and tissues [5].

In endodontics, calcium hydroxide was one of the first materials with bioactive characteristics (introduced in the 1920s) used to promote the formation of a dentinal bridge on exposed pulp tissue [6]. A few decades later, mineral trioxide aggregate (MTA) and its derivates were developed from the basic building material Portland cement, and, now, are commonly used in endodontics. These calcium silicate agents mainly include a mixture of Portland cement with bismuth oxide as an opacifier. The popularity of MTA-based materials in endodontics is due to their hydraulic nature, which confers to them the potential to set in a wet environment, such as root canals. These hydraulic cements, also known as bioceramics, are used for different clinical purposes such as vital pulp capping, perforation repair, apexification, apexogenesis, root canal filling, or as endodontic sealers [7,8]. The bioactivity of calcium silicate materials is a result of their potential to induce the formation of hydroxyapatite crystals on their surface [9]. Since the pH of hydraulic materials is high, phosphate ions from body fluids precipitate with the released calcium ions and form hydroxyapatite on the surface of the bioceramics [10].

Within the field of restorative dentistry, fluoride-releasing restorative material, such as glass ionomer, can be considered to be one of the first bioactive compounds, if we consider adhesion to dental tissues and release of fluoride as basis for bioactivity [11]. However, it should be noted that a bioactive material induces formation of hydroxyapatite on its surface; thus, bioactivity is not an ideal feature of restorative materials. Biomineralization properties of restorations can lead to calcium formation on the surface of dental materials. This aspect may play a positive role in the underlying dental tissue, since bioactive materials would inhibit the action of matrix metalloproteinase enzymes, and improve the hybrid layer. The most common bioactive materials used for restorative dentistry are either based on calcium silicate or calcium aluminate. Calcium silicate-based cements include Biodentine, which presents clinical indications similar to MTA, as well as potential to be used as an intermediate-stress restorative material, temporary restorative material and base/liner. Calcium aluminate restorative materials include direct restorative material and luting cements [12].

Concerning implantology, biaoactive materials have been used as coatings to improve the osseointegration of dental implants and enhance their overall biological performance [12,13]. Dental implants are made from bioinert materials such as stainless steel 316L, commercially pure titanium and its alloy Ti-6Al-4V, and cobalt–chromium alloys [14,15,16]. Different methods can be used to “coat” the surface of dental implants by bioactive materials, including enameling, sol–gel technique, electrophoresis, laser cladding, and thermal spraying. 45S5 Bioglass was the first bioactive glass, developed about 5 decades ago [15]. Other bioactive coatings include hydroxyapatite, zirconium dioxide, titanium dioxide, and zinc oxide. The characteristics of these materials can be further enhanced by adding active agents for different purposes. For instance, addition of silver ions to the bioactive glass structure may improve antibacterial properties [17].

In conclusion, research in the field of dental material is shifting from biocompatibility to bioactivity. In modern dentistry, the ideal dental material is not only biocompatible [18], but also provides biomimetic and bioactive properties. Different bioactive materials can be used in endodontics, restorative dentistry, and implantology and selection of the appropriate material strictly depends on the field of application and its properties.
